# Viscosified Solid Lipidic Nanoparticles Based on Naringenin and Linolenic Acid for the Release of Cyclosporine A on the Skin

**DOI:** 10.3390/molecules25153535

**Published:** 2020-08-02

**Authors:** Sonia Trombino, Camilla Servidio, Annarita Stella Laganà, Filomena Conforti, Mariangela Marrelli, Roberta Cassano

**Affiliations:** Department of Pharmacy and Health and Nutrition Sciences, University of Calabria, 87036 Arcavacata di Rende (CS), Italy; sonia.trombino@unical.it (S.T.); camillaservidi@gmail.com (C.S.); annaritalag@virgilio.it (A.S.L.); filomena.conforti@unical.it (F.C.); mariangela.marrelli@unical.it (M.M.)

**Keywords:** naringenin, linolenic acid, solid lipid nanoparticles, cyclosporine A, release, antioxidant, anti-inflammatory, skin

## Abstract

Psoriasis is one of the most common human skin disorders. Although its pathogenesis is complex and not completely know, the hyperactivation of the immune system seem to have a key role. In this regard, among the most effective systemic therapeutics used in psoriasis, we find cyclosporine, an immunosuppressive medication. However, one of the major problems associated with the use of cyclosporine is the occurrence of systemic side effects such as nephrotoxicity, hypertension, etc. The present work fits in this context and its aim is the design of suitable platforms for cyclosporine topical release in psoriasis treatment. The main objective is to achieve local administration of cyclosporine in order to reduce its systemic absorption and, consequently, its side effects. In order to improve dermal penetration, solid lipid nanoparticles (SLNs) are used as carriers, due to their lipophilicity and occlusive properties, and naringenin and linolenic acid are chosen, due to their properties, as starting materials for SLNs design. In order to have dermatological formulations and further modulate drug release, SLNs are incorporated in several topical vehicles obtaining gels with different degree of lipophilicity. Potential applications for psoriasis treatment were evaluated by considering the encapsulation efficiency, release profiles, in vitro skin permeation, and anti-inflammatory effects.

## 1. Introduction

Psoriasis is a multifactorial chronic inflammatory skin disorder that affects approximately 2 to 3% of the worldwide population. It is characterized by hyperproliferation and atypical differentiation of keratinocytes resulting in itchy, erythematous, and squamous plaques [1,2]. Psoriasis can present clinical features with a different degree of severity ranging from mild/moderate to severe manifestations with a large portion of cutaneous surface affected, negatively impacting the quality of life of the patient [3]. Moreover, psoriasis is associated with rheumatological, metabolic, and cardiovascular comorbidities [4].

The etiology of psoriasis is multifactorial and is considered to have an important genetic component. Regarding its pathogenesis, which is not completely elucidated, but the hyperactivation of the immune system seem to have a key role. In particular, several cell types are involved such as lymphocytes T, dendritic cells, and macrophages that infiltrate in dermis and epidermis and secrete proinflammatory mediators that stimulate, in their turn, the hyperproliferation of keratinocytes [5,6].

Current treatments include corticosteroids, keratolytics, phototherapy, calcineurin inhibitors, biologics targeting proinflammatory mediators and vitamin D analogues [7].

One of the most effective drugs approved for psoriasis treatment is oral cyclosporine A, an immunosuppressive medication. Its mechanism of action is attributable to the inhibition of calcineurin that consequently leads to the inactivation of T cells and downregulation of the expression of proinflammatory mediators, particularly IL-2 and IL-4 [8].

Although its proven efficacy, one of the major problems associated with cyclosporine is that its long-term use can cause systemic adverse effects such as hypertension, nephrotoxicity, hyperlipidaemia, etc. [9]. Therefore, cyclosporine topical administration would be advantageous. However, several attempts to achieve local administration of cyclosporine, by using numerous topical vehicles, have been made and failed [10,11,12,13]. This can be attributed to its unfavorable physicochemical properties for dermal penetration such as high molecular weight, low hydrophilicity and rigid structure [14,15,16].

In this regard, solid lipid nanoparticles (SLNs) have been widely investigated as potential topical delivery systems. In fact, their lipophilicity promotes topical drug penetration without damaging the skin barrier [17,18,19]. Moreover, SLNs show several advantages compared to other types of nanoparticles (i.e., polymeric and inorganic) such as biocompatibility, biodegradability, high loading efficiency, and easy scalability [20].

The aim of this work is the design of suitable platforms for cyclosporine topical release in psoriasis treatment. The main objective is to achieve site specific release of cyclosporine in order to reduce its systemic absorption and, consequently, its side effects. In order to improve dermal penetration, SLNs have been used as carriers and naringenin and linolenic acid were chosen due to their properties as starting materials for SLNs design.

Naringenin is a natural flavonoid exerting broad biological effects such as antioxidant, antibacterial, anti-inflammatory, and cardioprotective activities [21].

Interestingly, in vitro naringenin inhibited T-cells proliferation and reduced the levels of proinflammatory mediators such as TNF-alpha and IL-6 [22,23].

Linolenic acid is a polyunsaturated fatty acid belonging to omega-3 family. It shows potent anti-inflammatory activity attributable to the inhibition of TNF-alpha, inducible nitric oxide synthase and cyclooxygenase-2 [24]. Moreover, clinical studies reported that intravenous infusions of omega-3 fatty acids, in combination with oral supplementation, determined a reduction of psoriasis skin lesions [25].

For these reasons, in addition to the pharmacological activity of cyclosporine, the use of naringenin and linolenic acid could be useful in reducing the inflammatory state that intervenes in the pathogenesis of psoriasis.

In order to have dermatological formulations and further modulate drug release, SLNs have been incorporated in several topical vehicles such as Poloxamer 407, Carbopol and colloidal silica.

Potential applications for psoriasis treatment were evaluated by considering the encapsulation efficiency, release profiles, in vitro skin permeation, and anti-inflammatory effects.

## 2. Results and Discussion

### 2.1. Esterification of Naringenin with Linolenic Acid

**Scheme 1:**
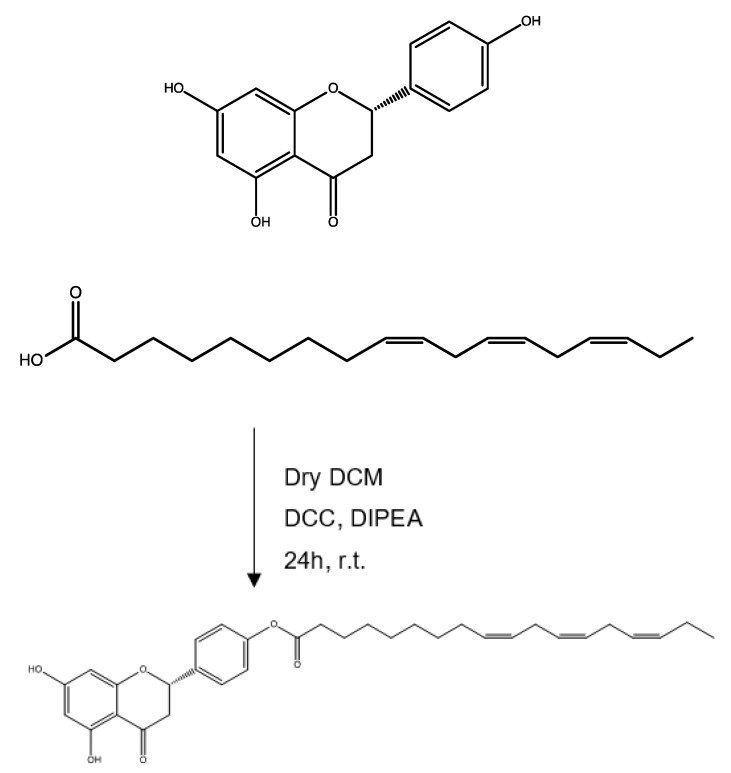
Ester synthetic route.

In order to obtain a lipophilic compound to use in the formulation of SLNs, the alcoholic group of naringenin was functionalized with the carboxylic group of linolenic acid. The esterification was carried out according Steglich reaction by using DCC as crosslinking agent in dry DCM at room temperature (Scheme 1) [26].

The product formation was confirmed by FT-IR and ^1^H NMR spectroscopy.

FT-IR (KBr) v (cm^−1^): 3550, 3200 (phenolic OH), 3079, 3028 (CH aromatic), 1731 (C=O).

^1^H-NMR (CDCl_3_): δ 1.09 (3H, t), 1.18–1.64 (8H, m), 1.90–2.15 (5H, m), 2.35–2.62 (4H, m), 2.60–2.68 (4H, m), 4.94 (1H), 5.29–5.38 (6H, m), 6.08–6.11 (2H, d), 7.06 (2H, m), 7.10 (2H, m). 

**Figure 1:**
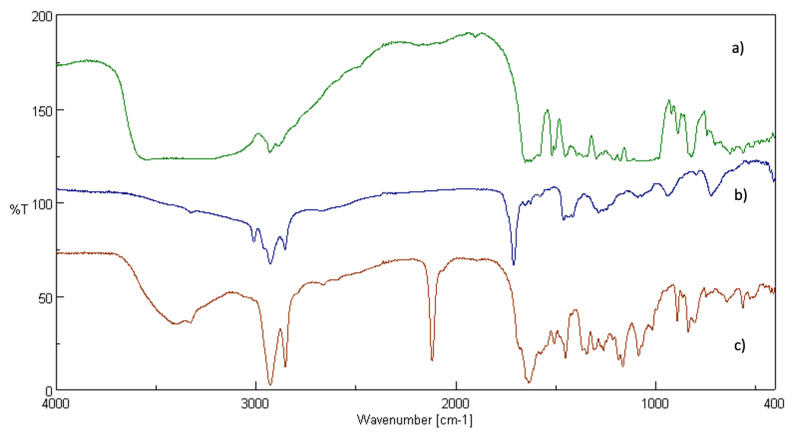
Fourier-transform infrared spectroscopy (FT-IR) spectra of: naringenin (**a**), linolenic acid (**b**), ester (**c**).

In particular, the FT-IR spectra of the ester (Figure 1c), naringenin (Figure 1b) and linolenic acid (Figure 1a) were compared.

The spectrum (Figure 1c) shows a new peak at 1731 cm^−1^, attributable to the stretching of the C=O of the ester, and peaks at 3079 to 3028 cm^−1^ and 3550 to 3200 cm^−1^, corresponding to the stretching vibrations of the phenolic OH and aromatic CH of naringenin, confirming the successful reaction.

Interestingly, on the base of NMR spectrum, it is possible to hypothesize a monoesterification of one of the two phenolic para-hydroxyl groups of naringenin.

### 2.2. Preparation and Characterization of SLNs

SLNs based on naringenin and linolenic acid and loaded with cyclosporin were prepared via microemulsion technique, obtaining an excellent encapsulation efficiency of about 92% [27]; this positive data can be attributable to the high lipophilicity of both Cyclosporine and SLNs. The encapsulation efficiency depends on several factors such as drug concentration, lipid matrix, etc. [28].

**Figure 2:**
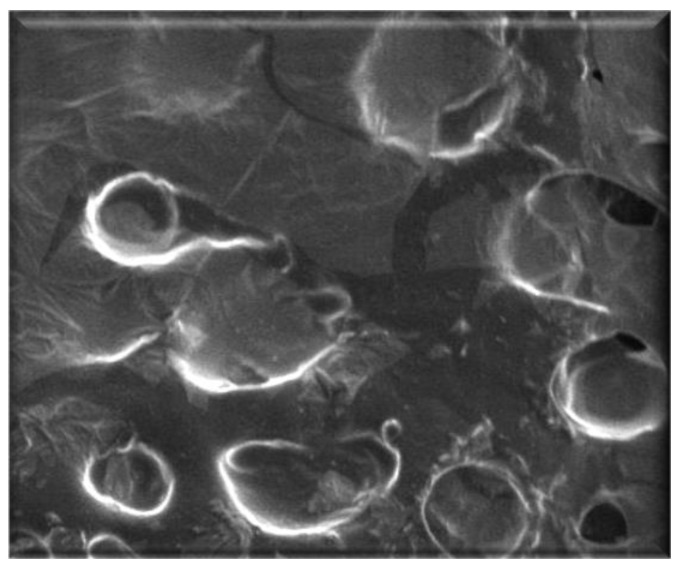
Photomicrography of loaded SLNs observed by SEM.

The obtained SLNs were characterized by morphological and dimensional analyses. DLS data show an average diameter of about 470 nm and PDI of 0.019, which is indicative of a good homogeneity in particle size distribution, and, moreover, confirm cyclosporine encapsulation considering the different average diameters of empty and loaded nanoparticles (Table 1). DLS results were confirmed also by SEM analysis that showed a spherical shape of SLNs (Figure 2).

### 2.3. Skin Permeation Studies

#### 2.3.1. In Vitro Permeation Studies

In order to have dermatological formulations and further modulate drug release, SLNs were incorporated in several topical vehicles such as functional substances such as hyaluronic acid and colloidal silica and gelling agents such as Poloxamer 407 and Carbopol, obtaining gels with a different degree of lipophilicity.

These dermatological formulations were subjected to transdermal release studies in order to evaluate their potential application and efficacy in psoriasis treatment.

Drug release profiles were evaluated by using Franz diffusion cells with cellulose acetate membranes or rabbit ear skin. Synthetic membranes, in addition to rabbit ear skin, were used due to the presence of cyclosporine that is characterized by a specific absorbance range (λ = 195–215 nm) and, therefore, can interfere with several skin components, such as lipid and proteins, that adsorb at similar wavelengths [29,30].

The presence of skin components in receptor chambers can be attributable to the release medium composition (0.9% NaCl/ethanol 20%), since ethanol can promote phospholipids mobility [31]. Ethanol was added to the release medium due to cyclosporine solubility in ethanol and not in water [32].

To further eliminate the risk of possible interferences, a 12kDa cut-off membrane was used.

In fact, the obtained data show, by comparing release studies carried out using rabbit ear skin and cellulose acetate membrane, an absence of significant interferences.

Drug release studies were performed on dermatological formulations at different time intervals (1, 2, 4, 8, 10, and 24 h). 

Drug release profiles were determined by UV-Vis spectrometry and expressed as percentage of the drug released in respect to the total loaded amount in function of time.

**Figure 3:**
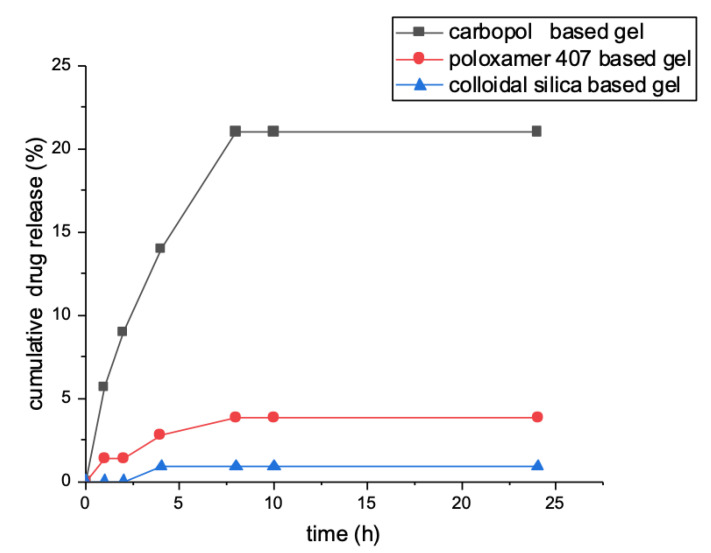
Cyclosporine release profile evaluated within 24 h.

Data showed that, with cellulose acetate membrane, cyclosporin A was released within 8 h from the dermatological formulations containing absorption enhancers in quantities ranging from 0.5 to 21% of the total loaded amount. After 8 h, no release was observed. Instead, cyclosporine was not released from SLNs and colloidal silica-based gel within 24 h (Figure 3).

**Figure 4:**
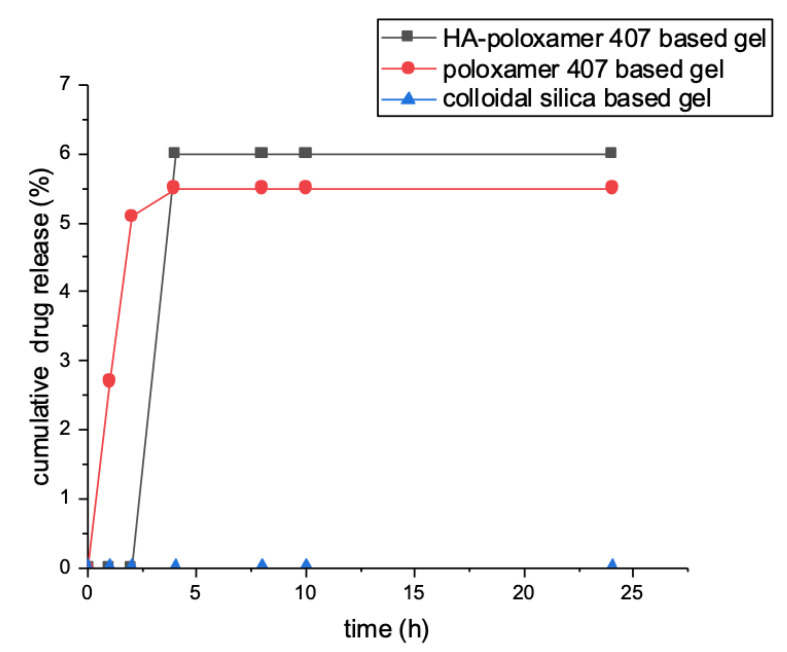
Cyclosporine release profile evaluated within 24 h.

On the other hand, by using rabbit ear skin, no drug release was observed, except in the case of gels containing absorption promoters, for which only a maximum release of 5% of loaded drug was reported (Figure 4).

A possible rationale for the obtained release profiles, showing no or low transdermal release, can be attributable to the use of SLNs as drug delivery system; in fact, SLNs are excellent drug carriers for dermal delivery but they show several limitations in transdermal delivery since they tend to remain in the superficial skin layers and not penetrate into deeper layers, increasing drug skin permeation thanks to their occlusive properties [33].

The addition of penetration enhancers in dermatological formulations, i.e., propylene glycol and transcutol P increases the solubility, permeation rate and skin retention of cyclosporine, thus leading to a low short-term transdermal release as observed for the Carbopol and Poloxamer 407 based formulations [34]. Moreover, this release behavior can be explained by hypothesizing that propylene glycol, in addition to increase drug solubility in skin layers, induces skin structural modifications, leading to a reduction of skin barrier properties and an increase of skin permeability, as reported in a recent study [35].

However, for all the dermatological formulations tested, no significative concentrations of cyclosporine were found in receptor chambers and, consequently, on the basis of these encouraging results, it is possible to hypothesize that these formulations may reduce or avoid systemic absorption of cyclosporine and consequently the occurrence of adverse effects.

#### 2.3.2. Tape Stripping Test

In order to evaluate the quantity of cyclosporin A released in the skin layers including the stratum corneum, epidermis, and dermis, the tape stripping method was performed after permeation studies.

The obtained data reported that the amount of the drug released within 24 h from the colloidal silica based gel is negligible in the stratum corneum and equal to 23% in the epidermis-dermis layer, showing a slower release rate, compared to the other dermatological formulations tested, most likely due to the absence of adsorption enhancers.

On the other hand, Poloxamer 407 based gel, released within 10 h, with approximately 79% and 15% of the loaded drug in the stratum corneum and epidermis-dermis layer, respectively, showing the most advantageous release profile. This data can be explained by the use, in addition to propylene glycol, of transcutol P as permeation enhancer, as it was proven to penetrate into the stratum corneum, thus enhancing drug solubility and retention into epidermis and decreasing the skin barrier functions [36].

Moreover, in addition to adsorption enhancers, the nature of the polymeric vehicle influences the release profile as well. In this regard, Carbopol-based gel showed a slower dermal release kinetic, compared to the Poloxamer-407 based gel, with approximately 36% and 28% of the loaded drug released, within 24 h, in the stratum corneum and epidermis-dermis layer, respectively. The faster release rate of the Poloxamer 407 based formulation can be attributed to its rapid dissolution in physiological conditions due to micellar disentanglement that occurs as result of water uptake. According to this, it can be assumed that, in this case, drug release is mainly driven by gel dissolution rather than diffusion [37].

Interestingly, by using Poloxamer 407 in combination with hyaluronic acid, a lower amount of the drug was released, with 15% in the stratum corneum and 12% in the epidermis-dermis layer after 10 h. This different release profile can most likely be attributable to an increased viscosity and strength of the gel, resulting in a more crosslinked structure with a consequent slower drug release rate [38]. In fact, the presence of hyaluronic acid led to the formation of a densely packed supramolecular structure, thus determining a reduction of the diffusion coefficient of the resulting gel [39]. In addition to this, the release behavior can also be determined in part by the retention of SLNs into the gel since, interestingly, recent modelling and experimental studies have shown that hyaluronic acid and phospholipids interact via hydrogen bonds and hydrophobic forces, thus leading to strong associations [40].

All the reported data suggest that the type and concentration of polymeric vehicle, crosslinker, adsorption enhancer and drug are the main determinant of release kinetics of topical formulations.

Furthermore, experimental results report a site-specific release of cyclosporine into skin layers, highlighting the therapeutic potential of these dermatological formulations in psoriasis treatment. This release behavior can be explained by the use of SLNs as drug carriers; in fact, due to their adhesive properties, SLNs tend to form films on the skin explicating occlusive properties. Due to the occlusion effect, SLNs induce enhanced skin hydration, by reducing water evaporation, thus leading to an increased drug penetration into skin layers. The occlusion factors are strongly influenced by several elements such as particle size, crystallinity of the lipid matrix, lipid concentration and mass [41,42,43,44].

Cyclosporine release from SLNs is determined by the combination of two different mechanisms: diffusion and lipid degradation [45]. In this regard, a recently reported model for diffusion release from lipid carriers based on Onsager’s theory can be considered as putative release mechanism for these delivery platforms. In particular, it is a self-healing, relaxation-dissolution mechanism on the basis of which it can be assumed that pores formation may be the result of random failures that occur due to the bending and surface tension of the lipid membrane. Therefore, when the system returns back to its equilibrium, a pores closure process driven by thermodynamic and hydrodynamic factors occurs [46].

### 2.4. Evaluation of Antioxidant Activity

SLNs and dermatological formulations ability to inhibit lipid peroxidation induced by tert-BOOH, a free radical generator, was examined in microsomal rat liver membrane. The antioxidant activity of the free ester was also investigated in the same conditions.

All the samples were able to preserve the antioxidant capacity of the precursor, naringenin, and, in particular, the most potent activity was exhibited by the ester, empty SLNs and HA-Poloxamer 407 based formulation; these last results can be attributable to the presence of hyaluronic acid that is also known to exert an antioxidant activity [47].

**Figure 5:**
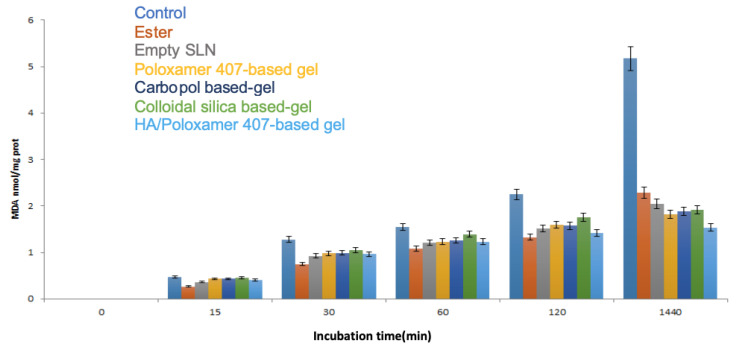
In vitro antioxidant activity.

However, the other formulations, even if not containing antioxidant substances, were able to preserve, in a dose dependent manner, microsomal membranes from lipid peroxidation induced by tert-BOOH, most likely due to the presence of naringenin and linolenic acid based SLNs (Figure 5).

### 2.5. Evaluation of Anti-Inflammatory Activity

**Figure 6:**
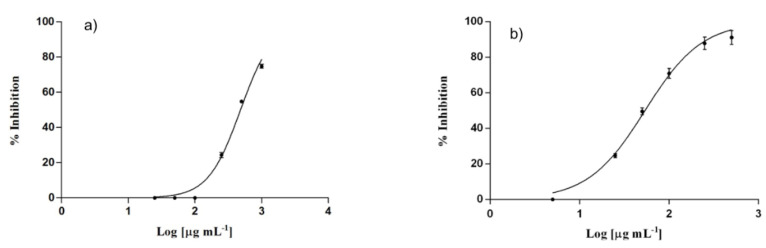
In vitro NO production inhibition of empty SLNs (**a**) and loaded SLNs (**b**).

Inhibition of nitroxide (NO) production by RAW 264.7 murine macrophage cell line, was assessed by determining the amount of nitrites, stable oxidized products of NO, after inducing an inflammatory stimulus by E. coli lipopolysaccharide (LPS). Obtained data (Figure 6) reported that both loaded and empty SLNs showed inhibitory activity against NO production in macrophages, when stimulated with LPS, with IC_50_ values of 53.78 ± 2.41 and 232.90 ± 11.7 μg/mL, respectively. In particular, loaded SLNs showed to be more active than empty SLNs, exhibiting a higher percentage of inhibition at a lower concentration. The greater anti-inflammatory activity of loaded SLNs is most likely due to the presence, in addition to naringenin and linolenic acid, of cyclosporin A, which is known to inhibit the production of nitroxide [48].

Interestingly, these results suggest that, SLNs, due to the presence of naringenin and linolenic, and cyclosporine could exert a synergistic anti-inflammatory activity in reducing the inflammatory state that intervenes in the pathogenesis of psoriasis, turning out to be promising cyclosporine delivery systems for psoriasis treatment.

## 3. Materials and Method

### 3.1. Chemicals

All solvents, analytical grade, were purchased from Carlo Erba Reagents (Milan, Italy): diethyl ether, dichloromethane (DCM), ethanol, methanol, acetone, dimethylformamide (DMF), 1-butanol, trichloroacetic acid (TCA), acetonitrile and n-hexane. Naringenin, linolenic acid, N,N-diisopropylethylamine (DIPEA), sodium sulfate, N, N-dicyclohexylcarbodiimide (DCC), sodium hydroxide, sodium taucholorate, polyoxyethylene sorbitan monolaurate (Tween 20), tert-butyl alcohol(TBA), butylhydroxytoluene (BHT) and ethylenediaminetetraacetic acid (EDTA) disodium salt dihydrate and phosphate buffer solution 1.0 M pH 7.4, DMEM, FBS, L-glutamine, penicillin/streptomycin, Griess reagent, L-NAME, and indomethacin were purchased from Sigma Aldrich (Sigma Chemical Co., St. Louis, MO, USA). Cyclosporine A was purchased from Farmalabor srl (Milan, Italy). RAW 264.7 cells were obtained from American Type Culture Collection (ATCC) no. TIB-71, UK.

Poloxamer 407, Carbopol 940, soy lectin powder, propylene glycol, isopropyl palmitate, nipagin, propyl paraben, colloidal silica, prunus amygdalus dulcis oil, ethoxydiglycol (Transcutol P). Cellulose acetate membrane (MWCO: 12,000–15,000 Da) was purchased by Medicell International LTD, London, UK.

### 3.2. Instruments

FT-IR spectra were measured using a Jasco 4200 IR spectrophotometer (Cremella (LC), Italy) with KBr disks. 1H-NMR spectra were recorded on a Bruker VM30 spectrometer (Milano, Italy); the chemical shifts were expressed as δ-values (ppm) and referred to the solvent. The UV–vis spectra were obtained using a Jasco UV-530 spectrophotometer (Cremella (LC), Italy) with quartz cells of 1 cm thickness. Scanning electron microscopy (SEM) analysis was performed with JEOL JSMT 300 A microscope (JEOL USA, Inc., 11 Dearborn Road, Peabody, MA, USA); the surface of the samples was made conductive by deposition of a thin gold layer in a vacuum chamber. Dimensional analysis of nanoparticles was carried out using a Brookhaven 90 Plus Particle Size Analyzer (Champaign, IL, USA) at 25 °C by measuring the autocorrelation function at 90° scattering angle. Sample were freeze-dried using Micro Freeze-drying Modulyo, Edwards. Dermatological formulations were prepared using Citounguator, Triad Scientifics (Memphis, TN, USA).

### 3.3. Esterification of Naringenin with Linolenic Acid

Esterification was carried out according to Steglich reaction.

In a three necked-flask equipped with a reflux condenser and dropping funnel, under stirring, accurately flamed and maintained under nitrogen bubbling, 0.2 g of naringenin (0.73 mmol), 0.288 g of DCC (1.4 mmol) and 0.126 mL of DIPEA (0.72 mmol) and then, after 30 min, 0.223 mL of linolenic acid (0.73 mmol) were dissolved in dry DCM. Then, DMF was added in order to increase the solubility of the compounds. The reaction mixture was kept under magnetic stirring for 24 h covered in foil. 

DCM was removed under reduced pressure while DMF with several extractions with diethyl ether and distilled water. Sodium sulfate was added to the organic phase, the solution was filtered, diethyl ether was removed under reduced pressure and the product was dried under vacuum obtaining a yellow solid. The compound was characterized trough FT-IR and ^1^H-NMR.

### 3.4. Preparation of SLNs

SLNs were prepared via microemulsion technique according to literature [49].

Briefly, the obtained ester, in the presence or not of cyclosporine A, was melted approximately at 70 °C. Meanwhile, a warm aqueous solution of Tween 20, sodium taurocholate, and butanol was prepared and added to the melt ester obtaining an O/A microemulsion. This microemulsion was then poured into a flask and kept under magnetic stirring at 2 to 3 °C in an ice bath for 45 min. Then, the two dispersions, of empty and loaded nanoparticles, were freeze-dried. For loaded SLNs, the unloaded drug was removed by filtration (Table 2).

The obtained samples were characterized trough dynamic light scattering and scanning electron microscopy. 

### 3.5. Encapsulation Efficiency Determination

The encapsulation efficiency of SLNs was determined by UV-Vis spectrometry. SLNs were dissolved in a water methanol solution (1:9) that was sonicated for 15 min at 37 °C in order to determine nanoparticles breaking and consequent drug release. The absorbance of the obtained samples was measured at a fixed wavelength for cyclosporine A (λ = 210 nm).

The encapsulation efficiency (EE%) is the percentage of loaded drug respect to the total amount of used drug and is calculated using the following Equation (1),

(1) $$\mathrm{EE}\%=\frac{Ql}{Qt}\times 100$$

where *Qt* indicates the total amount of drug used and *Ql* the loaded drug amount.

Studies were carried out in triplicate and the results were in agreement with ±5% standard error.

### 3.6. Preparation of Poloxamer 407-Based Gel

0.09 g of propylene glycol was solubilized in 6.12 g of distillated water and, then, 1.28 g of Poloxamer 407 were added under vigorous stirring. The obtained gel was left in the refrigerator overnight. Separately, 1 g of soy lecithin was emulsified with 1 g of isopropyl palmitate and left to rest for 24 h. Subsequently 0.004 g of loaded SLNs were suspended in a solution of transcutol P that was mixed with lecithin solution and Poloxamer gel with Citounguator for 2 min.

### 3.7. Preparation of Carbopol-Based Gel

To prepare, 0.09 g of propylene glycol and 0.1 g of Carbopol were solubilized in 9.29 g of distilled water containing 0.01g of disodium EDTA.

The formation of the gel occurred after the addition of sodium hydroxide (solution 10%) up to pH 6. Subsequently, 0.04 g of loaded SLNs were suspended in a transcutol P solution that was mixed with the gel with Citounguator for 2 min.

### 3.8. Preparation of Colloidal Silica-Based Gel

To prepare, 0.01 g of BHT was dissolved in 8.9 g of sweet almond oil and, then, to the resulting solution 0.004 g of loaded SLNs were added. The obtained solution was mixed with anhydrous colloidal silica with Citounguator for 4 min.

### 3.9. Preparation of Hyaluronic Acid and Poloxamer 407-Based Gel

To prepare, 0.01 g of hyaluronic acid and 0.004 g of loaded SLNs were solubilized in 5 mL of bidistilled water. Then, 3 g of Poloxamer 407 were added under mixing and the resulting gel was sonicated for 1 h.

### 3.10. In Vitro Skin Penetration Studies

Skin penetration studies were performed (*n* = 8) by using franz diffusion cells apparatus with cellulose acetate membranes and rabbit ear skin (furnished from local butcher) for 24 h. The apparatus was maintained at 36.5 °C in order to mimic physiological conditions. Receptor chambers (6.0 mL) were filled with NaCl 0.9% solution containing ethanol (20%) and kept under stirring in order to maintain sink conditions. Unloaded SLNs alone or in dermatological formulations were used as control.

At specific time intervals (1, 2, 4, 8, 10, and 24 h) an aliquot (1 mL) of each sample was withdrawn from receptor chambers and replaced with fresh release medium. Samples were analyzed trough UV-Vis spectrophotometry and drug release profiles were expressed as percentage of drug released respect to the total loaded amount in function of time.

### 3.11. Tape Stripping Test

After permeation studies, samples were removed from skin surface. Rabbit ear skin were washed three times with phosphate buffer (pH = 7.4) and then dried. Subsequently, the stratum corneum was separated from dermal layers (epidermis and dermis) using an adhesive tape (Scotch 845 Book Tape, 3M). Previous studies reported that 15 strips are enough to separate dermal layers [50]. Cyclosporine A was extracted from adhesive tapes by vortexing them with acetonitrile for 2 min. The resulting solution was then filtered with 0.45 μm membrane and the amount of cyclosporine was determined by UV-Vis spectrophotometry.

On the other hand, the epidermis-dermis layer was cut into small pieces, vortexed for 5 min with 1 mL of acetonitrile and then sonicated for 40 min. Subsequently, the resulting solution was filtrated with 0.45 μm membrane and the amount of cyclosporine A was determined by UV-Vis spectrophotometry.

### 3.12. Malonoaldehyde Assay

One mL of microsomal suspension was added to an ethanolic solution containing 0.07 mL of BHT 0.2%, 3 mL of TCA a 0.5% and 0.5 mL of TBA. The samples were then incubated with the microsomal suspension in a thermostatic bath at 37 °C for 24 h. At specific time intervals (15 min, 30 min, 1 h, and 24 h) the samples were withdrawn, heated at 90 °C and then centrifugated. Subsequently, the thiobarbituric acid-malondialdehyde complex was detected spectrophotometrically at λ = 535 nm.

### 3.13. In Vitro Anti-Inflammatory Activity Evaluation

The anti-inflammatory activity of both empty and loaded SLNs was investigated in vitro, assessing their ability to inhibit NO production in RAW 264.7 cell line. Dulbecco’s modified Eagle’s medium (DMEM) supplemented with L-glutamine, fetal bovine serum, and a solution of penicillin and streptomycin (1%, 10%, and 1%, respectively) was used as cell medium. Cells were cultured at 37 °C under 5% CO_2_ and were seeded onto microplates (96 wells, 100,000 cells/well). After 24 h, the medium was removed and fresh medium containing samples at different concentrations and 1 μg/mL LPS were added. After 24 h of incubation, 100 μL of Griess reagent was added in order to evaluate the presence of nitrite, a stable product of NO oxidation. The presence of nitrite was determined spectrophotometrically at λ = 490 nm. Indomethacin and L-NAME were used as positive controls.

### 3.14. Statistical Analysis

Data are expressed as mean (standard error of the mean of N replicates per experiment). Statistical analysis was carried out by Student’s *t*-test using the GraphPad Prism 4 software program. *p* < 0.05 was considered as statistically significant. 

## 4. Conclusions

The aim of this work was the design of suitable platforms for cyclosporine topical release in psoriasis treatment. The main objective was to achieve site specific release of cyclosporine in order to reduce its systemic absorption and, consequently, its side effects. In order to improve dermal penetration, SLNs were used as carriers and naringenin and linolenic acid were chosen as starting materials for SLNs design due to their anti-inflammatory activity. SLNs with excellent loading efficiency and size suitability for topical administration were prepared via microemulsion technique. In order to have dermatological formulations and further modulate drug release, SLNs were incorporated in several topical vehicles such as functional substances, such as hyaluronic acid and colloidal silica, and gelling agents such as Poloxamer 407 and Carbopol, obtaining gels with a different degree of lipophilicity. Therefore, the cyclosporine release profile was evaluated, and in vitro data showed a site-specific release of cyclosporine in skin layers and a low transdermal release. Furthermore, SLNs showed the potent in vitro anti-inflammatory activity turning out to be useful in reducing the inflammatory state that intervenes in the pathogenesis of psoriasis. In conclusion, the obtained results showed that these formulations, thanks to their biocompatibility, release profiles, excellent antioxidant and anti-inflammatory activities could be promising systems for Cyclosporine topical release.

## Figures and Tables

**Table 1:** DLS analysis.

Formulation	Average Diameter (nm)	PDI
Empty SLNs	299.9 ± 3.1	0.274 ± 0.013
Loaded SLNs	470.0 ± 4.6	0.195 ± 0.047

**Table 2:** Amount of reagent used in SLNs preparation.

Ester	Tween 20	Butanol	Sodium Taurocholate	Cyclosporine A
0.05 g	0.032 mL	0.012 mL	0.016 g	0.001 g
